# Intergenerational Continuity of Intimate Partner Violence Perpetration: An Investigation of Possible Mechanisms

**DOI:** 10.1177/0886260520959629

**Published:** 2020-09-25

**Authors:** Sania Shakoor, Delphine Theobald, David P. Farrington

**Affiliations:** 1 Queen Mary University of London, United Kingdom; 2 Kingston University, London, United Kingdom; 3 University of Cambridge, Cambridge, United Kingdom

**Keywords:** intimate partner violence, intergenerational transmission, psychosocial factors

## Abstract

Intimate partner violence (IPV) is a continuum of abuse that is associated with a number of negative outcomes including substance misuse, depression, and suicidal ideation. This study aims to investigate the intergenerational transmission of IPV perpetration and the mechanisms involved. Intergenerational transmission was investigated using information from two generations of the Cambridge Study in Delinquent Development which is a prospective longitudinal study of 411 males from an inner London area in the UK who have been followed up over a period of 50 years. Information with regard to IPV perpetration, specifically physical violence, was garnered from self-reports by the male at age 32, from their female partner at age 48, and from their male and female children in early adulthood. Regression analyses were used to investigate intergenerational transmission and examine whether psychosocial risk factors could be identified as potential intergenerational pathways. Having a father who was a perpetrator of IPV significantly increased the odds of daughters being perpetrators by 2 times. It did not significantly increase the odds for sons. The intergenerational transmission of IPV perpetration remains between fathers and their daughters over and above a series of psychosocial factors such as accommodation problems and alcohol misuse. Identification of factors associated with the intergenerational transmission of IPV perpetration will inform practitioners and policymakers. Information garnered from studies such as this may contribute to the development of prevention and intervention strategies for those at risk.

## Introduction

Intimate partner violence (IPV) is closely associated with the more commonly used term “domestic violence” but is often considered to include a wider range of contexts and behaviors specifically with regard to partners in dating, cohabiting, and marital relationships (refer to Corvo, 2019).  IPV can be conceptualized as a continuum of abuse, including homicide, minor and severe physical assault, sexual assault, psychological abuse, including threats, harassment, coercion, and intimidation (World Health Organization, 2013). This type of violence can occur in all types of intimate relationships and can vary in type (e.g., severe versus less-severe), frequency (e.g., regular occurrence versus rare), and purpose (e.g., instrumental versus reactive; Klostermann & Fals-Stewart, 2006, p. 588).  Various studies as well as meta-analyses suggest that both men and women can act as perpetrators and victims of IPV (Archer, 2000; Dutton, 2007; Stith et al., 2000; Straus, 2011; Theobald & Farrington, 2012) and may result from the normalization of violence as a way to manage conflict (Raghavan et al., 2006; Raiford et al., 2013). IPV is increasingly reported and is thus a huge burden for public health as it occurs regardless of age, socioeconomic status, gender, and sexuality, and it can result in a number of negative outcomes. These can include substance misuse, post-traumatic stress disorder, depression, and suicidal ideation (Simmons et al., 2015) and this list is not exhaustive.

### Intergenerational Transmission

Not least, and perhaps of greatest concern, is the possibility of intergenerational transmission of IPV. The relationships within families, particularly those between parents and children, are highly influential, and life-course events, drug and alcohol abuse, and IPV can have huge impact on their lives (Thornberry et al., 2003). This intergenerational transmission is a widely studied explanation of how family characteristics can impact on the development of aggression and violence in adult relationships (Herzberger, 1996). Children reared in households where they are exposed to acts of violence may in turn learn that violence is an appropriate reaction in interpersonal conflictual situations and act accordingly in their own relationships in adulthood (Franklin & Kercher, 2012). There is currently robust evidence to support this proposition, but, there are inconsistencies (refer to Stith et al., 2000; Stith et al., 2004). These authors suggest that children may have differential responses to this experience, with some studies suggesting an increase in their perpetration, some suggest an increase in their victimization, whereas others suggest that children fare as well as those not exposed (Kitzmann et al., 2003). This might be explained by the heterogeneity of exposure and experiences (Simons et al., 1998; Kalmuss, 1984). These outcomes may also be moderated by gender, as some studies suggest that girls often respond to IPV by internalizing the trauma and become victims in future relationships, whereas boys tend to externalize the trauma and are more likely to become perpetrators of IPV subsequently (Doumas et al., 1994; Gover et al., 2008). However, other studies have found that such children, regardless of gender, report significantly higher rates of perpetration when compared with controls (White & Widom, 2003). The findings across studies will depend on the design of the study, the demographics of the population surveyed, the measurements used, and the duration of the follow-up.

There is currently a dearth of studies that use multiple waves of data garnered from prospective longitudinal studies that address the limitations of prior research. These limitations include the use of cross-sectional or short-term follow-up studies, male-only studies, female-only studies, and measurement anomalies (Knight et al., 2016). Knight and colleagues investigated both the perpetration and victimization of IPV and found a stronger association for intergenerational transmission for female offspring. Consequently, they have called for further exploration of gender differences in intergenerational transmission, separately for perpetration and victimization.

### Contributory Risk Factors

It is important to note that there is evidence to suggest that children exposed to family violence may fare as well as those not exposed (Kitzmann et al., 2003), highlighting the need to identify factors that may act as protective and risk factors in shaping intergenerational transmission of IPV. Current evidence suggests that a number of factors increase the vulnerabilities for being involved in IPV as either victims or perpetrators. Psychosocial factors such as exposure to multiple adversities, where children are exposed to violence, maltreatment, low self-esteem, and socioeconomic disadvantage (Capaldi et al., 2012; Knight et al., 2016; Stith et al., 2000; Theobald & Farrington, 2012; White & Widom, 2003) have all been found to be associated with individual variations in IPV. Whether there is a direct relationship between any one of these factors and IPV is unclear, and it is likely that these factors may have interactive or sequential effects. Furthermore, there is some evidence to suggest a genetic risk, with heritability estimates ranging between .15 and .54 for victimization and perpetration of IPV (Barnes et al., 2013; Hines & Saudino, 2004). This genetic propensity may be translated to the offspring of IPV perpetrators and victims through the environments they provide (gene-environment correlation), which in turn may heighten the risk of intergenerational transmission of IPV. It is thus beneficial to take such psychosocial factors into consideration when investigating the intergenerational transmission of IPV.

The current study aims to add to the limited literature on intergenerational transmission using data from the Cambridge Study in Delinquent Development (CSDD: refer to Farrington et al., 2006), a prospective longitudinal study that has followed a complete cohort of males, recruited at age 8, for over 50 years, where two generations have reported on perpetration of IPV. A considerable amount of information has been collected over time, and information about intimate relationships was collected at ages 32 and 48 from the males and their partners. This allows for an investigation into the intergenerational transmission of IPV perpetration as the children of these original males transition into adulthood. Information is derived from male and female reports using the Conflict Tactics Scale (CTS; Straus, 1979) in both generations. The main aims of this article are to determine if there is a relationship between paternal and offspring perpetration of IPV and to investigate what psychosocial risk factors may contribute to this association. Findings may inform the design and implementation of prevention and intervention policies.

## Method

### Ethics

The male and female biological children of the original men were contacted and interviewed between 2004 and 2013 after gaining agreement from their parents as specified by the South–East Region Medical Ethics Committee. All participants gave informed consent at the beginning of the interviews.

### Design and Participants

The CSDD is a prospective longitudinal survey of 411 boys who were originally living in an urban working-class area of South London, UK (refer to Farrington et al., 2006; Farrington et al., 2009; Farrington et al., 2013). The original boys (generation 2 or G2) constituted a complete population of boys aged 8–9 who were attending 6 primary schools in the area in 1961–1962. Twelve boys from a local school for educationally subnormal children were included in the sample in an attempt to make it more representative of the population of boys living in the area. The boys were followed up through face to face interviews up until age 18 through to age 48 and with the exception of interviews at age 21 and 25 the majority of those still alive were interviewed, at age 14, 405 (99%) were interviewed, 399 (97% at age 16, 389 (95%) at age 18, 378 (94% at age 32 and 365 (93%) at age 48. The boys were predominantly working class, from two-parent households where the majority were white (87%) having parents where both were born and brought up in the UK or Southern Ireland, however, a small proportion (3%) has at least one parent of West Indian or African origin, at least one parent from Cyprus, or from another country, that is, Australia, France, Germany, Malta, Poland, Portugal, Spain, and Sweden (refer to Farrington, 1995, 2003). Their fathers’ employment was mainly unskilled and semi-skilled manual work in 93.7% of cases, which was higher than the national average at that time of 78.3%.

### Procedure

#### G2: Males and partners.

When the G2 males were interviewed at age 32, they reported whether there had been violence in their relationship, including hitting their partner with no retaliation, their partner hitting them with no retaliation, and both partners hitting the other. Forty-two men were involved in physically violent relationships with their partner at this age (Farrington, 1994). In the age 48 interviews a more comprehensive breakdown of the types of violence in relationships was gathered using the CTS (Straus, 1979) based on the partner’s report (refer to Theobald & Farrington, 2012). We then combined the age 32 and age 48 reports (i.e., IPV occurring at either age 32 or 48) and found that 208 (65.2%) of the 319 men who were known at both ages had committed no violence at either age. This left 111 (34.8%) reports of IPV; 32 (10.0%) men had hit with no retaliation, and there were 40 (12.6%) cases where the man and the woman were both involved in the perpetration of violence. Therefore, 72 (22.6%) men committed IPV at either age 32 or age 48, compared with 247 non-violent men. One third (32%) of the violent men at age 32, compared with 16% of the non-violent men, were still violent at age 48. An odds ratio (OR) of 2.4, although not statistically significant (because of lack of power), was substantial and suggests the continuity of male IPV across the 16 years between ages 32 and 48. This age 32–48 combined data are the measure of IPV perpetration in this article.

#### G3: Male and female children.

All G3 male and female biological children of the G2 men were targeted for interviews between 2004 and 2013 at a minimum age of 18 (born up to 1995). Of the 653 eligible G3 children, 551 were interviewed (84.4%) at an average age of 25, including 291 of the 343 G3 males (84.8%) and 260 of the 310 G3 females (83.9%).

### Measures

The CTS (Straus, 1979) is a measure of IPV and was used when interviewing the G2 partners at age 48 and the G3 males and G3 females. Its format allows the interviewer to ask questions about the occurrence of IPV in the last 5 years. It includes reciprocal questions on verbal abuse and minor, moderate, and serious acts of physical violence (e.g., Has he done it to you? Have you done it to him?). As we were interested in a measure of physical violence, which was concordant with actual physical assault or serious threat, we only included items which captured more serious acts in our measure of violence, namely, slapping, shaking, throwing an object at, kicking/biting or hitting with a fist, hitting with an object, twisting arms, throwing bodily, beating up (multiple blows), choking or strangling, and threatening with or using a weapon. Although the CTS has limitations (refer to Archer, 1999), it is considered to be a reliable and valid instrument to measure IPV across different populations (Straus, 2004).

#### Psychosocial risk factors.

Information that was originally gathered when the G2 male was aged 48, measuring life success (refer to Farrington et al., 2006) was used. Four composite factors were; satisfactory accommodation (i.e., whether they were a homeowner, the housing was of good quality and whether they had moved less than 3 times in the last 5 years); satisfactory employment history (i.e., whether they were currently unemployed, not of low social class, had reasonable take-home pay and no long periods of unemployment in last 5 years); satisfactory alcohol use (i.e., not driven whist under the influence of alcohol, not a heavy drinker, not a binge drinker and a low score (0–1) on the CAGE questionnaire (Mayfield et al., 1974); no drug use (i.e., not taken cannabis, or other drugs). A further variable was also included, namely, father getting into fights at age 32 (refer to Auty et al., 2015).^1^1Further developments in mediation analyses have shown that indirect effects between a set of variables can exist in the absence of significant direct effects in both of the component paths (refer to Hayes, 2009).  Three variables based on G3 offspring interviews were also identified as potential markers of IPV perpetration; coming from a disrupted family (father left the family home before the child’s sixteenth birthday), poor parental supervision (parents never know where their child was going when they went out before age 16) and harsh parenting (parents hit their children with an implement as a form of discipline).

### Analytic Strategy

Relationships between the variables were investigated using logistic regression models in Stata version 14.1 statistical software. Participants in this study were fathers and their offspring, which resulted in non-independent observations. All analyses were thus adjusted for dependence based on the sandwich or Huber–White variance, which adjusts estimated standard errors (Williams, 2000).

**Figure 1.**
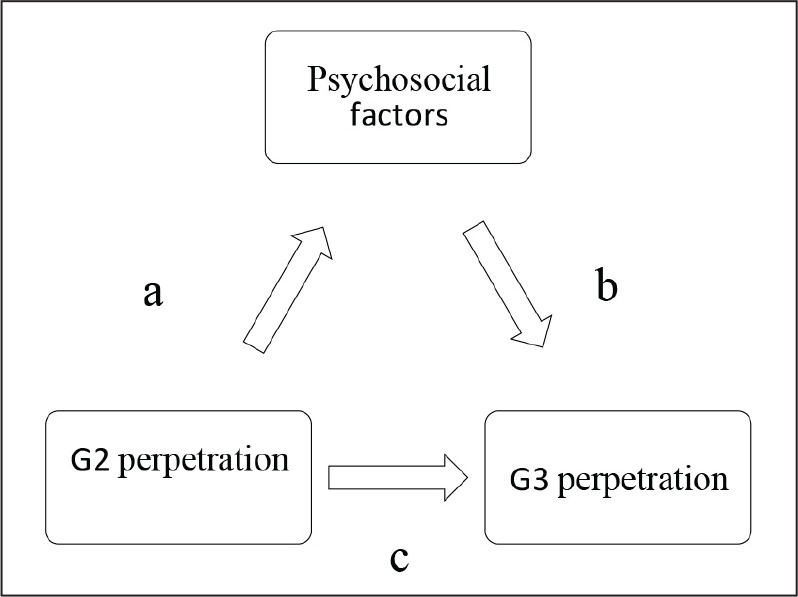
Theoretical illustration of Baron and Kenny’s mediation model.

*Note*. This model aims to identify the mechanisms which underly the observed relationship between the independent variable (G2 perpetration) and the dependent variable (G3 perpetration) via the inclusion of third hypothetical variables (Psychosocial factors).

Further to investigate bivariate relationships between the phenotypes of interest, G3 paternal and G2 offspring psychosocial factors were included in multiple logistic regression models as covariates to investigate the proportion of covariance they explained between G2 and G3 IPV perpetration. A total of 90% confidence intervals were used because clear directional predictions justified one-tailed statistical tests. Our analyses were based on the Baron and Kenny (1986) mediation framework (refer to Figure 1), which explores the role of psychosocial factors as mediating mechanisms through which G2 IPV perpetration translates into an increased risk of G3 IPV perpetration (Baron & Kenny, 1986). We conducted these analyses in a series of steps:

Step 1: Test for a direct effect between the independent variable (G2 IPV perpetration) and the dependent variable (G3 IPV perpetration);
Step 2: Test for a direct effect between the independent variable (G2 IPV perpetration) and psychosocial risk factors (mediating mechanisms);
Step 3: Test for a direct effect between psychosocial risk factors (mediating mechanisms) and the dependent variable (G3 IPV perpetration); and
Step 4: Test to see if direct effect between the independent variable (G2 IPV perpetration) and the dependent variable (G3 IPV perpetration) remains when controlling for psychosocial risk factors (mediating mechanisms). Step 4 analyses were only performed in the presence of a direct effect between G2 IPV perpetration and G3 IPV perpetration (Figure 1—Path c) and where the psychosocial factor was significantly associated with either G2 IPV or G3 IPV (Figure 1—Path a or b).

## Results

### Association between G2 Paternal and G3 Offspring Intimate Partner Violence

Among G3 males, 37.6% had a G2 father who perpetrated IPV and 17.13% were IPV perpetrators themselves; 32.39% of G3 females had a G2 father who perpetrated IPV and 32.1% were IPV perpetrators themselves. G3 females were almost twice as likely to be perpetrators as G3 males (refer to Theobald, Farrington, Ttofi, et al., 2016). 

**Table 1.** Association between Paternal and Offspring Intimate Partner Violence Perpetration.

	Prevalence of IPV Perpetration		Association between Generation 2 and Generation 3 IPV
	Generation 2 % (Total *N*)	Generation 3 % (Total *N*)	Odds ratio (90% CI)
Generation 3			
Total sample	35.26 (312)	24.55 (497)	1.88 (1.13, 3.16)
Males	37.65 (170)	17.13(251)	1.77 (.81, 3.89)
Females	32.39 (142)	32.11 (246)	2.28 (1.23, 3.77)

*Note*. Analyses are adjusted for age of G3 (Median age = 25.0, Interquartile range 22.9–27.8).

Using logistic regression models we explored whether paternal G2 perpetration of IPV was associated with G3 offspring perpetration of IPV. The results indicated that having a G2 father who was a perpetrator of IPV did not significantly increase the odds of being a perpetrator of IPV among G3 males (OR = 1.77, CI, .81, 3.89). Having a G2 father who was a perpetrator of IPV increased the odds of being a perpetrator by two times among G3 females (OR = 2.28, CI, 1.23–3.77). This suggests that there was evidence of intergenerational transmission for IPV perpetration only among female offspring (Table 1).^2^2Cohen (1996) suggests that an OR greater than 2 is a large effect size.

### Relationship between G2 Perpetration of IPV, G3 Perpetration of IPV and Psychosocial Risk Factors

Analyses from logistic regression models indicated that G2 perpetration of IPV was significantly associated with a series of psychosocial disadvantages, including greater risk of accommodation problems, employment problems, and drug use (Tables 2a and 2b—Step 2). For G3 offspring, having a G2 father who perpetrated IPV significantly increased the odds of belonging to a disrupted family (Male: OR = 7.81, CI 2.18, 28.05; Female: OR = 4.62, CI 1.47–14.58). Having a G2 father who perpetrated IPV significantly increased the odds for experiencing poor supervision for G3 female offspring only (OR = 1.96, CI 1.05–3.67); and receiving harsh parenting (OR = 3.70, CI 1.99, 6.87) for G3 male offspring only.

Factors associated with socioeconomic background and substance use among G2 males were significant antecedents of G3 offspring perpetration of IPV (Tables 2a and 2b—Step 3). G2’s experiences of accommodation problems (OR = 2.55, CI 1.32–4.91) and getting into fights (OR = 2.31, CI 1.14–4.71) increased their G3 male offsprings’ odds of IPV perpetration by approximately 2–3 times. G2’s experiences of employment problems were significantly associated with an approximate 2–4.5 times increased odds of G3 male (OR = 4.51, CI 2.46–8.27) and G3 female (OR = 2.40, CI 1.32–4.38) offspring perpetrating IPV. Having a father who reported alcohol misuse increased the likelihood of being a perpetrator of IPV among G3 female offspring only (OR = 2.03, 90% CI 1.07–3.88).

Analyses investigating G3’s individual psychosocial factors demonstrated that belonging to a disrupted family did not significantly predict G3 male perpetration but did predict female IPV perpetration (Tables 2a and 2b—Step 3). Both G3 males (OR = 1.86, CI 1.05–3.31) and G3 females (OR = 2.18, CI 1.40–3.41) who experienced poor parental supervision were more likely to become perpetrators of IPV.

**Table 2a.** Intergenerational Risk of IPV Perpetration: The Role of Paternal and Offspring Psychosocial Factors among Male Offspring.

Step	Psychosocial Factors	Odds Ratio (90% CI)	*B* (*SE*)	Proportion of Total Variance Explained
1	G2 –> G3	1.77 (.81, 3.89)		–
2	G2 -> Accommodation problems	3.87 (1.17, 12.88)	1.35 (.73)	–
	G2 -> Employment problems	2.26 (.82, 6.23)	.82 (.62)	–
	G2 -> Alcohol misuse	1.89 (.78, 4.59)	.64 (.54)	–
	G2 -> Drug use	5.27 (1.44, 19.29)	1.66 (.79)	–
	G2 -> Fights	3.47 (1.23, 9.76)	1.24 (.63)	–
	G2 -> Disruptive family	7.81 (2.18, 28.05)	2.06 (.78)	–
	G2 -> Poor supervision	1.68 (.97, 2.89)	.52 (.33)	–
	G2 -> Harsh parenting	3.70 (1.99, 6.87)	1.31 (.38)	–
3	Accommodation problems -> G3	2.55 (1.32, 4.91)	.93 (.40)	–
	Employment problems -> G3	4.51 (2.46, 8.27)	1.51 (.37)	–
	Alcohol misuse -> G3	1.00 (.54, 1.85)	–.01 (.37)	–
	Drug use -> G3	1.68 (.89, 3.17)	.52 (.39)	–
	Fights -> G3	2.31 (1.14, 4.71)	.84 (.43)	–
	Disrupted family -> G3	1.65 (.92, 2.93)	.50 (.35)	–
	Poor supervision -> G3	1.86 (1.05, 3.31)	.62 (.35)	–
	Harsh parenting -> G3	1.39 (.80, 2.42)	.33 (.34)	–

*Note*. Analyses adjusted for age of Generation 3; G2 = Generation 2 paternal IPV perpetrations; G3 = Generation 3 offspring IPV perpetrations cf= Controlling for; CI= Confidence interval; SE = Standard error.

Please note that as a non-significant main effect was observed between G2 perpetrators and their male offspring, Step 4 of the analyses was not conduced for male offspring.

**Table 2b.** Intergenerational Risk of IPV Perpetration: The Role of Paternal and Offspring Psychosocial Factors among Female Offspring.

Step	Psychosocial Factors	Odds Ratio (90% CI)	*B* (*SE*)	Proportion of Total Variance Explained
1	G2 -> G3	2.28 (1.23, 4.25)	0.83 (0.38)	–
2	G2 -> Accommodation problems	3.43 (1.31, 9.01)	1.23 (.59)	–
	G2 -> Employment problems	5.62 (1.93,16.40)	1.73 (.65)	–
	G2 -> Alcohol misuse	1.34 (.46, 3.91)	.30 (.65)	–
	G2 -> Drug use	1.93 (.58, 6.40)	.66 (.73)	–
	G2 -> Fights	1.86 (.65, 5.31)	.62 (.64)	–
	G2 -> Disruptive family	4.62 (1.47, 14.58)	1.53 (.70)	–
	G2 -> Poor supervision	1.96 (1.05, 3.67)	.67 (.39)	–
	G2 -> Harsh parenting	1.23 (.56, 2.72)	.21 (.48)	–
3	Accommodation problems -> G3	1.66 (.89, 3.10)	.51 (.38)	–
	Employment problems -> G3	2.40 (1.32, 4.36)	.88 (.37)	–
	Alcohol misuse -> G3	2.03 (1.07, 3.88)	.71 (.39)	–
	Drug use -> G3	1.08 (.60, 1.95)	.08 (.36)	–
	Fights -> G3	1.41 (.76, 2.64)	.35 (.38)	–
	Disrupted family -> G3	1.69 (1.04, 2.74)	.53 (.29)	–
	Poor supervision -> G3	2.18 (1.40, 3.41)	.78 (.27)	–
	Harsh parenting -> G3	1.35 (.83, 2.19)	.30 (.30)	–
4	G2 -> G3 (cf Accommodation problems)	1.88 (1.03, 3.45)	.63 (.37)	.24
	G2 -> G3 (cf Employment problems)	1.86 (.96, 3.60)	.62 (.40)	.25
	G2 -> G3 (cf Alcohol misuse)	2.14 (1.14, 4.03)	.76 (.38)	.08
	G2 -> G3 (cf Disrupted family)	2.29 (1.17, 4.50)	.83 (.41)	.00
	G2 -> G3 (cf Poor supervision)	2.07 (1.09, 3.92)	.73 (.39)	.12
	G2 -> G3 (cf Harsh parenting)	2.28 (1.23, 4.24)	.83 (.38)	.00

*Note*. Analyses adjusted for age of Generation 3; G2 = Generation 2 paternal IPV perpetrations; G3 = Generation 3 offspring IPV perpetrations cf= Controlling for; CI= Confidence interval; SE = Standard error.

Tables 2a and 2b show the four steps proposed by Baron and Kenny (1986) to test for possible mediators of the intergenerational transmission of IPV from G2 to G3.

Step 1 shows the intergenerational transmission from G2 IPV to G3 IPV

Step 2 shows whether G2 IPV is related to the psychosocial risk factors

Step 3 shows whether these psychosocial risk factors are related to G3 IPV

Finally, Step 4 shows whether the intergenerational transmission remains when controlling for the risk factors.

### Do Paternal and Offspring Psychosocial Factors Contribute towards the Intergenerational Transmission (G2 to G3) of Intimate Partner Violence Perpetration?

As a non-significant main effect was observed between G2 perpetrators and their male G3 offspring, Step 4 of the analyses was not conduced for male offspring (Table 2a). Among G3 females, the overall multiple regression analyses demonstrated that the intergenerational risk of IPV perpetration remained after controlling for a series of paternal and offspring psychosocial factors, with exception to employment problems. In our sample employment problems explained 25% of the covariation between G2 and G3 IPV perpetration, with G2 fathers; IPV perpetration no longer remaining a significant risk factor for their G3 daughters IPV perpetration (Table 2b—Step 4).

## Discussion

The main aims of this study were first to determine if there was a relationship between paternal and offspring perpetration of IPV and second to investigate what psychosocial risk factors contribute to this association. The findings add to the literature and support what has been reported elsewhere. With regard to the first aim, our findings support the literature in so far as girls who experience violence between their parents have a high^2^ likelihood of being perpetrators themselves (Smith et al., 2011). A total of 32% of G3 girls committed IPV compared with 17% of boys. This finding supports previous findings in the CSDD (refer to Theobald, Farrington, Ttofi, et al., 2016) and Archer (2002) which suggests that females are more often perpetrators of IPV but possibly at the less severe end of the continuum of physical abuse, for example, using slapping or pushing. It is important to note that although women may perpetrate violence equally or more so than men, the complexity of contributory factors may differ and women’s use of violence should be viewed within the context of intersectionality factors, including gender, class, race, social and civil opportunities (White & Kowalski, 1994). It is acknowledged that although mixed, there is some evidence to suggest that females can more often be the victims when coercive control and physical violence are considered (Carney, & Barner, 2012) and may become perpetrators of IPV as a means of self-defense (Swan et al., 2008). The intergenerational association was only significant for females (OR = 2.28). This is in line with Knight et al. (2016) who found a stronger intergenerational effect for females than for males.

Importantly, the findings suggest that psychosocial factors that have previously been associated with the perpetration of IPV were also found in this sample, but that there were differential effects across gender. For example, having a father who reported alcohol misuse increased the likelihood of female offspring perpetrating IPV. Also, coming from a disrupted family did not significantly predict male offspring perpetration of IPV. but did predict for daughters. However, poor parental supervision was a significant predictor of IPV perpetration for both male and female offspring. The occurrence of IPV in the family of origin will depend on a number of interacting contextual, social, biological, psychological, and personality factors (Klostermann & Fals-Stewart, 2006, p. 589).

Even though there appears to be a consensus that violence generally is often associated with alcohol and/or drug use, it may be an indirect rather than a direct relationship. This may imply that it is not necessarily the substance abuse itself but the quality of the environment that is affected in terms of relationship quality, which results in conflict and IPV, and it is these factors that may impact on the transmission from one generation to the next. With regard to the impact of a disrupted family on the perpetration of IPV for the female offspring, it is worthy to note that the G2 males were born in the early 1950s when family breakdown was not common so the analyses may be influenced to some extent by the proportions of females who experienced this. So, replication would be necessary to investigate the generalizability of these findings to more contemporary samples where there are higher rates of family breakdown (i.e., separations, and children being taken into care). Clearly, there are other interacting variables that were not included in our analyses, for example, personality factors such as antisociality which may be genetically transmitted across generations (Thornberry et al., 2003).

Regarding possible contributory mechanisms, the findings suggest that for daughters the intergenerational risk of IPV perpetration remained over and above a series of paternal and offspring psychosocial factors. Therefore, exposure to paternal IPV may contribute directly to the learned belief that violence is an acceptable way of conflict resolution (Akers & Sellers, 2009), thus increasing their risk of IPV perpetration over and above psychosocial risk factors (e.g., low socioeconomic background). Interestingly our finding that the intergenerational risk of IPV perpetration does not remain significant once employment problems are taken into consideration suggests that paternal employment problems may contribute vulnerabilities of IPV perpetration which go beyond the risk posed by the exposure to paternal IPV itself. This is in line with the notion that girls are more susceptible to their environment and therefore exposure to adverse environments such as paternal unemployment may exasperate their risk of IPV perpetration (Silverthorn, & Frick, 1999). Consequently, focusing on environmental factors associated with paternal employment problems such as financial strain and poor life satisfaction may be of importance when supporting individuals at risk of IPV perpetration.

As IPV is in part heritable (Barnes et al., 2013; Hines & Saudino, 2004), it is possible that the genetic propensity to IPV is, in part, being transmitted through the environment provided by the fathers. This exploration of passive gene-environment correlation (Jaffee & Price, 2007) was not within the remit of this study and is an area for future studies to explore, utilizing genetically sensitive study designs (e.g., the twin methodology). Furthermore, there is increasing evidence to suggest that exposure to childhood adversities (i.e., violence in the home) can result in epigenetic mechanisms to be involved in the biological embedding of early life experiences (Romens et al., 2015), which may consequently increase vulnerabilities for IPV. Moreover, substance misuse, accommodation, and employment problems may be markers for a number of individual differences such as psychopathology (refer to Auty et al., 2015), and maybe a cause and a consequence of marital discord, poor parenting practices, poor parental supervision, and drug and alcohol dependence. These factors can be associated with the strain of financial difficulties, which can lead to an accumulation of problems that may impact on the family in a negative way. It is important that these factors are investigated in other prospective longitudinal studies that have data available to allow further understanding of the mechanisms involved in the intergenerational perpetration of IPV.

### Strengths/Limitations and Future Implications

The strengths of this research are that we were able to investigate the intergenerational continuity of IPV based on male and female reports of G2 male perpetration and on G3 male and female offspring reports utilizing the same measure of IPV. It is also important that we did not exclude G3 children based on the status of the parental relationship or their own relationship. Some studies exclude individuals who are not married, which may underestimate the level of IPV perpetration, as much higher IPV rates have been found in dating couples and cohabiting couples than in married couples (e.g., DeKeseredy & Schwartz, 1998; Theobald, Farrington, Ttofi, et al., 2016). Also, Archer (2002) found that young women were less likely to report perpetration in community samples rather than in other samples (e.g., student). The age of the respondent and the source of reporting will undoubtedly impact on the results and findings may well be confounded with socioeconomic disadvantage in some groups.

This study is not without limitations. Although most studies on IPV utilize versions of the CTS, with some researchers using only parts of the questionnaire, we report on moderate and more severe violence perpetration. This outcome variable was dichotomized, so information about the frequency, severity, and variability of IPV within the perpetrator group may be lost. However, dichotomization is often used because the frequency distributions of IPV and violence generally are highly skewed, and correlational analyses are not appropriate because parametric and distributional assumptions are not fulfilled (Farrington & Loeber, 2000; Straus, 1979). Dichotomization allows for the OR to be calculated, which represents an effect size that is easily understood by policymakers (refer to Farrington & Loeber, 2000). We also acknowledge that the CTS, whilst being the most widely used for measuring the prevalence of both perpetration and victimization of IPV (Archer, 1999; Langhinrichsen-Rohling, 2005), has its limitations. It relies heavily on the frequencies of violence and does not take contextual factors such as chronicity of abuse and fear of the victim into consideration. Thus, what may appear to be symmetry across genders in prevalence may represent differential experiences (i.e., women may be more negatively impacted; Stark, 2010). However, one of the strengths of the present study when measuring intergenerational transmission of IPV the same measurement tool was used. The aim of the study was to calculate the risk of intergenerational transmission and identify potential mechanisms, which the CTS has allowed us to do. The next step for researchers would be to utilize qualitative methods to explore the intricacies of these relationships. Lastly, our measure of IPV focused on overt physical forms of IPV, it is therefore important to interpret our findings of intergenerational transmission within this context and for further studies to explore this risk within wider dimensions of IPV.

There are some important implications for these findings, as the identification of possible factors associated with the intergenerational transmission of IPV will inform both practitioners and policymakers. The information garnered from studies such as this (especially regarding mediating factors), will help the development of targeted intervention strategies for those most at risk. These can include early intervention with families who are experiencing financial hardship through unemployment and associated factors, such as drug and alcohol abuse and psychopathology. Further research should carry out similar studies investigating the intergenerational transmission of being a victim of IPV as well as a perpetrator. It would also be of interest, when considering successful interventions, to better understand whether intergenerational transmission includes family only perpetration or a general violent tendency by the perpetrator (refer to Theobald, Farrington, Coid, et al., 2016).

## Author Biographies

**Sania Shakoor**, PhD, is a lecturer in Mental Health at Queen Mary, University of London. She is a chartered psychologist who completed her PhD in Social, Genetic and Developmental Psychiatry at Kings College London. Dr. Shakoor’s overarching research interests lie within the development of mental health problems (i.e., psychotic experiences) among individuals exposed to interpersonal violence across the lifespan (i.e., bullying, intimate partner violence). To date, her work has utilized genetically sensitive longitudinal data from large cohorts to explore underlying mechanisms (i.e., environmental adversities, cognitive processing, etc.) that shape the mental health of children and adolescents.

**Delphine Theobald**, PhD, is an associate professor of Forensic Psychology at Kingston University. Her main areas of research are in life-course transitions and offending behavior, intimate partner violence, and psychopathology associated with violence perpetration. More specifically her work examines the effects of marriage, cohabitation, and parenthood on offending behavior, and the antecedents of violence perpetration. Recently her interests have widened to include aggression in dating relationships and the evaluation of “healthy” relationship interventions. In 2013, she received the Early Career Award from the Division of Developmental and Life-course Criminology of the American Society of Criminology for her significant contribution to scholarly knowledge on developmental and life-course criminology in her early career.

**David P. Farrington**, OBE, is emeritus professor of Psychological Criminology at the Institute of Criminology, Cambridge University. He is a Chartered Forensic Psychologist whose interests focus particularly upon developmental and life-course criminology, longitudinal studies of criminal careers, risk-focused prevention, and evaluating the effectiveness of interventions. He is the director of the Cambridge Study in Delinquent Development, which is a 50-year follow-up of 400 London males. Professor Farrington has published widely on risk factors and the development of offending, methodological quality in evaluation research, comparative criminology, crime and physical health, criminal career models, shoplifting, bullying, developmental prevention, CCTV, street lighting, risk assessment, desistance, cost-benefit analysis, the effectiveness of programs for young offenders, psychopathy, developmental and life-course theories, offender profiling, and the prediction of homicide.
